# Risk of What and Why? Disaggregating Pathways to Extremist Behaviours in Individuals Susceptible to Violent Extremism

**DOI:** 10.1002/bsl.2710

**Published:** 2024-12-08

**Authors:** Caitlin Clemmow, Nicola Fowler, Amber Seaward, Paul Gill

**Affiliations:** ^1^ Department of Security & Crime Science UCL London UK; ^2^ Prevent In‐Place Team Birmingham & Solihull Mental Health NHS Foundation Trust Birmingham UK

**Keywords:** radicalisation, risk assesment, structured professional judgement, terrorism, violent extremism

## Abstract

Best practice in violent extremist risk assessment and management recommends adopting a Structured Professional Judgement (SPJ) approach. The SPJ approach identifies relevant, evidence‐based risk and protective factors and requires experts to articulate hypotheses about a) what the person might do (risk of what), and b) how they've come to engage in the concerning behaviour (and why) (Logan 2021) to inform who, needs to do what, and when. Whilst the field continues to move towards adopting an SPJ approach, there remains a gap between what is known empirically and what is needed in practice. We apply psychometric network modelling to a sample of 485 individuals entered into Channel, the UK's preventing and countering violent extremism (P/CVE) program. We model the system of interactions from which susceptibility to violent extremism emerges, providing data driven evidence which speaks to risk of what and why. Our research highlights a way to generate evidence which captures the multifactorial nature of susceptibility to violent extremism, to support professional decision making in the context of an SPJ approach.

## 1 Introduction

Best practice in violent extremist risk assessment and management recommends adopting a Structured Professional Judgement (SPJ) approach (Borum 2015a, 2015b; Monahan 2017). The SPJ approach identifies relevant, evidence‐based risk and protective factors and requires experts to articulate hypotheses about (a) what the person might do (**risk of what**), and (b) how they've come to engage in the concerning behaviour (**and why**) (Logan 2021). A good formulation articulates **risk of what and why** to inform bespoke case management plans to reduce risk and wider harm (Logan, Nathan, and Brown 2011). Whilst Preventing and Countering Violent Extremism (P/CVE) continues to move towards adopting an SPJ approach to managing violent extremist risk, there remains a gap between research and practice.

Research on violent extremism mainly deals with single or a handful of risk factors and their individual effects on a single outcome, most often extremist violence. However, practice must contend with far more complexity, formulating hypotheses about a range of different extremist behaviours (e.g., violence, radicalising others, travelling to a foreign conflict zone, extreme group membership, etc.). Risk and protective factors may be more or less relevant, or have different functional roles, across these different outcomes. Formulating extremist risk also requires considerations of many complex and mutually reinforcing interactions between the different risk factors potentially driving the behaviour—something that the average regression model often used within science does not provide. As such, a new approach to research is needed to deliver the science that practice needs.

We aim to bridge this gap by applying methodologies from complexity science to model the system of interactions from which susceptibility to violent extremism emerges, providing data driven evidence that speaks to risk of what and why in a sample of cases managed by the UK's P/CVE program, Channel. We demonstrate a method capable of evidencing the SPJ approach to violent extremist risk assessment and management, disaggregating different extremist behaviours and some of the many multifactorial pathways to different concerning behaviours. In the following section we introduce the rationale for the present study, reviewing the literature on risk of what, and why, before introducing the present study.

## 2 Background

### 2.1 Risk of What?

The risk assessment process should be clear on the outcome of concern—the **risk of what**—it is trying to assess (Borum 2015b; Roberts and Horgan 2008; Logan 2021). However, violent extremists are not a homogeneous group (Ellis et al. 2016; Gill, Horgan, and Deckert 2013), and involvement in violent extremism is not a single phenomenon, as radicalisation has multiple potential outcomes (Borum 2015b; Logan and Lloyd 2019; Perliger, Koehler‐Derrick, and Pedahzur 2016; Schuurman 2020). Violent extremism comprises numerous violent and nonviolent individual activities, including ideological extremism, fundraising, recruitment, logistical support, attack planning, and violence. Indeed, most individuals convicted of terrorism offences are involved with financial or other nonviolent activities (Hodwitz 2021; Horgan et al. 2016). Some have worked to develop theoretical and empirical typologies of these roles, primarily distinguishing between ideological support, material facilitation, and physical involvement (Borum 2015a; Horgan, Shortland, and Abbasciano 2018; Simcox and Dyer 2013; Taylor and Horgan 2006).

However, research has historically been limited by focusing primarily on violent extremism as a single dependent variable, when this should be disaggregated (Gill and Horgan 2013; Gill and Young 2011; Horgan, Shortland, and Abbasciano 2018; LaFree et al. 2018). Some recent disaggregation work has compared violent and nonviolent outcomes of radicalisation, finding empirical differences in: social relationships, personal experiences, and self‐beliefs (Knight, Woodward, and Lancaster 2017, 2022); social control and social learning factors (Becker 2021; LaFree et al. 2018); and perceived loss of significance (Jasko, LaFree, and Kruglanski 2016). Even within single ideologies, violent and nonviolent individuals differ in age (Gill and Horgan 2013; Klausen, Morrill, and Libretti 2016), online posting behaviours (Scrivens et al. 2023), TRAP‐18 indicators (Challacombe and Lucas 2018), radicalising settings and behaviours (Bartlett and Miller 2012), and socio‐political beliefs (Kerodal, Freilich, and Chermak 2016).

Fewer studies have gone beyond this binary violence distinction to compare across multiple role types and activities. These have mostly centred on sociodemographic differences: between Palestinian suicide bombers and those involved in other terrorist roles in the United States (Gill and Young 2011); different PIRA activities (Gill and Horgan 2013); frontline and suicide operatives within ISIS foreign fighters (Evans, Milton, and Young 2021); individuals involved in Islamist‐inspired violent extremism varying in violence of activity, training abroad, and speed of progressing through the organisation (Perliger, Koehler‐Derrick, and Pedahzur 2016); behaviours prompting referral to the UK's Prevent program (Seaward et al. 2024); and Horgan et al.'s (2018) actor‐supporter‐facilitator typology. Currently, research has not yet distinguished between roles and activities on the basis of behavioural and psychological indicators, rather than merely sociodemographic variables. It is these observable and actionable indicators that may provide value to practice, allowing development of risk assessment approaches that distinguish between individuals at risk for different extremist outcomes that require different urgency and P/CVE resources (Knight, Woodward, and Lancaster 2017). Of equal importance is understanding the drivers or pathways to different violent extremist behaviours—that is, understanding why.

### 2.2 And Why?

Understanding the drivers—in other words, why some individuals move towards engaging in violent extremism—is also key. Several conceptual models of radicalisation theorise why some come to engage in extremism. Most describe individual‐level causal mechanisms that underpin trajectories towards violent extremism (Borum 2003; Moghaddam 2005; Neo 2019; Precht 2007; Sageman 2008; Silber, Bhatt, and Analysts 2007; Wiktorowicz 2004). Some present multi‐level conceptual models (Bouhana 2019; McCauley and Moskalenko 2017; Taylor and Horgan 2006; Veldhuis and Staun 2009).

In general, these models conceptualise susceptibility to violent extremism as emerging from the interaction between individual‐level susceptibilities, situational exposures to violent extremism (people places, settings), and triggering events. For instance, McCauley and Moskalenko's (2008) two‐pyramid model outlines mechanisms of political radicalisation as they describe pathways to extremist violence. Whilst such models provide a framework for understanding why some engage in extremism, research suggests that ‘fully observing a complete framework in practice is uncommon’ (Bouhana and Schumann 2022).

Moreover, practice routinely deals with observable indicators—imperfect proxies for more complex processes driving concerning behaviour. These observable behavioural indicators are often prioritised over more abstract and difficult to objectively observe theoretical mechanisms. Further compounding the issue, empirical studies have now uncovered hundreds of correlates of radicalisation and violent extremism, offering little guidance on how to operationalise this knowledge for practice.

Much of this research has now been summarised by a series of systematic reviews and meta‐analyses (Gill, Clemmow, et al. 2021; Hassan et al. 2018; Wolfowicz et al. 2021). Whilst a tangible demonstration of the progress of the field, long lists of correlates speak little to why some individuals come to engage in concerning behaviour, and this understanding is needed to inform an SPJ approach to violent extremist risk assessment and management.

More recently, research has begun to evidence the multi‐ and equi‐finality of pathways to violent extremism, further underscoring the complexity of why some move towards violent extremism (Clemmow, Bouhana, and Gill 2020; Clemmow et al. 2023; Corner, Bouhana, and Gill 2019; Gill, Farnham, et al. 2021). Some more complex research designs examine how a handful of risk factors come together to impact on violent extremist outcomes. For instance, studies examine pathways to violent extremism driven by misogyny (Rottweiler and Gill 2021), conspiracy beliefs (Rottweiler and Gill 2020), exposure to online material (Pauwels, Brion, and De Ruyver 2014), mental disorder and psychological distress (Corner and Gill 2019), psychological vulnerabilities (Borum 2014), and more, demonstrating why some individuals, in some instances, move towards violent extremism.

However, such studies are limited in that they only examine the few factors relevant to their research question. Practice, particularly a formulation‐based approach to risk assessment and management, routinely deals with far more complexity than a handful of risk factors. Hypothesising why an individual is presenting concerning behaviour involves careful consideration of the evidence base to formulate why a particular behaviour is occurring, and therefore how to manage that risk. Much of this decision‐making is based on years of expertise and often clinical experience. To date, little attempt has been made to empirically model this complexity. However, we suggest that it is possible to do just so, to provide an empirical evidence base to guide expert decision‐making and further evidence the adoption of an SPJ approach to violent extremist risk assessment and management—this is the aim of the present study.

### 2.3 The Present Study

We apply methods from complexity science to model the multifactorial relationships between risk factors for different extremist behaviours, in a sample of individuals deemed susceptible to violent extremism, who had been accepted onto the Channel program—the P/CVE arm of the UK's counterterrorism strategy. We apply psychometric network analysis, a clustering algorithm, and shortest path analysis to model the many different mutually reinforcing interactions among risk factors present in the case files of just under 500 people presenting some sort of concern. We then analyse how these relate to different concerning behaviours—preaching, radicalising others, extreme group membership, financial support of terrorism, attack planning, and extremist action. We highlight patterns of risk factors associated with different outcomes (risk of what), and articulate different routes to a range of concerning behaviours (and why). The present study therefore asks the following research questions.

1. Risk of what: What clusters of risk factors are present in a sample of individual susceptible to violent extremism, and how are these related to different extremist outcomes?
2. Why: What are the different routes to and between these outcomes?

We discuss our results in terms of evidence for an SPJ approach to violent extremist risk assessment and management.

## 3 Method

### 3.1 Sample

The sample includes 497 individuals who had been accepted onto the Channel programme in England and Wales between 2012 and 2014. The Channel program is a multi‐agency process which aims to identify and prevent susceptible people engaging in violent extremism. It requires consent from the individual and is therefore voluntary. Individuals accepted at Channel would have been assessed by a range of professionals as presenting some sort of concern about susceptibility to violent extremism and/or engaging in terrorism. If the individual is assessed as not meeting the threshold for the Channel program, the case would not be adopted, and would not be taken forward for intervention.

It's important to note that Channel is an upstream, preventative arm of the UK's counterterrorism strategy. The goal is to identify susceptible people, early, and intervene by meeting unmet needs before criminal action occurs. In doing so, the aim is to reduce the potential for harm to society by reducing the pool of **potential** criminal actors. Most of these individuals would likely not go on to commit a violent terrorist act but rather are susceptible to being drawn down a path towards violent extremist action.

Table 1 summarises the descriptive statistics of the sample. Notably, the sample includes more children than would be expected of a cohort engaging in criminal action (see Supporting Information S1: Figure S1 for the age distribution). The justification for including children is that these children were assessed by professionals as susceptible to being drawn into violent extremism. This does not necessarily mean that they posed a risk of committing terrorist violence, but that there was a concern about susceptibility in need of intervention. The concern could originate from contact with a family member previously convicted of a terrorist offence, or because of concerning statements being made at school.

**TABLE 1:** Sample descriptive statistics (*n* = 485).

	Min	Max	Mean	SD
Age	6	60	23.61	10.3
Unknown	65			

	*n*	%
Gender		
Male	444	91.3%
Female	39	8.0%
Unknown	2	0.0%
In school/training/employed		
Yes	224	46.2%
No	166	34.2%
Unknown	95	19.6%
Ethnicity		
White	155	32.0%
Black	27	5.6%
Asian	165	34.0%
British	3	0.6%
Caribbean	1	0.2%
Mixed race	8	1.6%
Other	17	3.5%
Unknown	109	22.5%
Place of birth		
Im(migrant)	64	13.2%
British born	1	0.2%
British born (second generation)	195	40.2%
Grandparents/parents born in Britain	2	0.4%
Illegal immigrant/no leave to remain	1	0.2%
Unknown	222	45.8%
Ideology		
Islamist	170	35.1%
Extreme Right wing	102	21.0%
Extreme Left wing	4	0.8%
Single issue	4	0.8%
Other	8	1.6%
Unclear/not reported	197	40.6%

Excluding these cases from the sample would not be justified given that a proportion of the Prevent cohort is increasingly made up of children and young people (32% of referrals in 2023 were 15–20 years old, 31% were 14 and under; Home Office 2023), and recent events highlight that some do go on to engage in violent extremism. However, it's important to note that this sample is not generalisable to terrorist offenders, and any findings must be limed to understanding risk in individuals in the upstream P/CVE space, only.

Each of the nine regions of England & Wales (North West, North East, South West, South East, Wales, West Midlands, East Midlands, Eastern, London) submitted case files to be analysed by practitioners at Birmingham and Solihull Mental Health Foundation Trust. Data for 12 cases were missing and so those individuals were removed the sample. The final sample size was *n* = 485.

### 3.2 Procedure

The present study is a secondary analysis of data collected by a team of practitioners delivering front‐line services under the UK's counterterrorism strategy—Prevent. Our research team was granted access to the closed‐source data. The following section summarises the original research methodology carried out by the Birmingham and Solihull Mental Health Foundation Trust's Vulnerability Support Hub team. Practitioners from the Trust applied Patton's (2002) analytical steps for qualitative data to generate the coding framework.

1. Prepare the data.
  All referrals were obtained electronically and anonymised, accessed only on Birmingham and Solihull Mental Health Foundation Trust premises by the researchers involved in the study who held sufficient security clearance. The raw data consisted of case files which included various documents related to the individual's referral, vulnerability assessment, and intelligence profile. These documents contain closed‐source information collated and verified (as much as possible) by professionals working in CVE and included information from police, health, statutory services, and other agencies involved in delivery of the Channel program.
2. Define the unit of analysis.
  The original research team defined the unit of analysis as the individuals case files.
3. Develop a coding framework.
  Inductive coding was applied to qualitative case files to extract all features present in an initial small sample of cases. Rather than being theory or previous‐research driven, this type of framework development methodology is data‐driven and exploratory, with the aim being to generate novel insights to inform subsequent theory development and/or hypothesis testing in confirmatory research designs (Patton 2002). The result of the process was a draft coding framework.
4. Test your coding framework on a sample of cases.
  Two researchers independently coded a unique sample of cases to identify any new codes and test the interrater reliability of the draft coding framework. Any new codes that emerged were integrated into the draft coding framework and existing codes were refined where interrater reliability was less than optimal. This was an iterative process until saturation was achieved (no new codes emerged), and high interrater reliability (> 0.70) was achieved.
5. Code all of the text.
  The final coding framework was applied to all remaining case files.
6. Assess coding consistency.
  All cases were double coded by two independent researchers. Disagreements were resolved by consensus. Where consensus could not be reached a third independent coder reviewed all of the available materials and made a final decision. Inter‐rater reliability was assessed, throughout, with high inter‐rater reliability achieved (> 70%).

The resulting database consists of risk factors and behaviours identified in the case files of 485 Channel participants.

### 3.3 Measures

Table 2 summarises the factors identified by the original team of researchers submitted for secondary analysis to the present study. The factors broadly cover demographics, diagnoses and symptoms of mental illness, forensic history, past supervision and intervention failures, vulnerability, risk to self, risk to others, family and relationship problems, identity issues, early maladjustment, history of traumatic experiences, grievance, substance use problems, social networks, and negative and pro‐criminal attitudes. All factors were operationalised as a binary variable indicating presence (= 1) or absence (= 0). All variables were entered into network models as independent variables.

**TABLE 2:** Descriptives statistics for all factors identified in the case files of 485 individuals accepted to the Channel program.

Theme	Variable	Description	Frequency	%
Active psychological symptoms	Dysregulated behaviours & emotions	Unmanageable/intense emotions, sexually disinhibited behaviour, deliberate self‐harm	163	33.6%
	Psychosis	Delusions, hallucinations, thoughts disorder, paranoia, erratic behaviour, mood disorders, mania	114	23.5%
	Depression	Low mood, sleep problems, hopelessness, negative view of self/other/future, low self‐esteem, poor motivation	96	19.8%
	Anxiety	Avoidance, high arousal, hypervigilance, OCD	56	11.5%
	Symptoms of trauma	Fear, high emotions, flashbacks, nightmares, avoidance	48	9.9%
	Anger	Angry outbursts, difficulty controlling anger	164	33.8%
Attitudes	Extreme views	Extremist views and/or beliefs	224	46.2%
	Them versus us thinking	Demonising or dehumanising the 'other' group	159	32.8%
	Endorses violent groups	Approves of or sanctions violent groups	125	25.8%
	Pro‐criminal attitudes	Disrespect for police officers, disregard for the law, justification for crimes	102	21%
	Negative attitudes to authority	Distrusts, questions, actively resists directions and rules set by authority figures or people in positions of power	106	21.9%
	Endorses violence	Approves of or sanctions the use of violence	174	35.9%
Coping styles	Poor coping skills	Maladaptive/poor coping skills (avoidance, isolation, etc)	66	13.6%
	Chaotic life	Drug and alcohol problems, unstable home, risky situations, etc	68	14%
	Substance abuse problems	Regular dependent drug or alcohol use which has a significant impact on functioning and well‐being, or it results in negative relationship, legal or social consequences (e.g. debt)	140	28.9%
	Threats of violence	Stated threats to commit violence	228	47%
Criminality	Violent offending	Actual violence or threatening behaviours likely to result in psychological harm, weapons/firearms offences	179	36.9%
	Non‐violent offending	Non‐violent offending against property, theft and kindred offences, fraud	177	36.5%
	Hate crime	Violent or non‐violent crime driven by racism or prejudice	41	8.5%
	ASB	Anti‐social behaviour including delinquency, ASBOs, breach of the peace, drunk and disorderly)	87	17.9%
	Previously imprisoned	Custodial sentence	79	16.3%
	Capability (for extremist violence)	Assessed as demonstrating capability to engage in extremist violence	192	39.6%
Current stressors	Financial problems	Recent financial problems (debt, loss of income, etc)	41	8.5%
	Recent victim	Recent victim of crime, assault, prejudice, threats, etc (violent or non‐violent)	47	9.7%
	Transitional period	Period of intense change and/or transition, deployment, divorce, relocation	216	44.5%
	Currently being exploited	Currently being exploited i.e. by peers, gang, county lines, etc	58	12%
	Housing issues	Experiencing homelessness, lack of suitable housing, etc	105	21.6%
Extremist behaviour	Attack planning	Behaviour towards a terrorist attack such as reconnaissance, weapon procurement, etc	37	7.6%
	Financial support of extremism	Sending or saving money to support extremism	5	1%
	Member of extreme group	Member of an extreme group	132	27.2%
	Radicalising others	Attempting to or actively radicalising others	27	5.6%
	Preaching	Expressing extreme views publicly	112	23.1%
	Extremist action	Committing extremist action such as violence, hate crime, etc	45	9.3%
Grievance	Perceived injustice	A challenge to someone's beliefs about a ‘just world’	151	31.1%
	Excluded	Not able to access things that other people do, not included, not able to fit in or be accepted	26	5.4%
	Prejudice	Attack, negative experiences or discrimination on an ethnic or cultural basis	41	8.5%
	Perceived threat	Perceptions of threats from others, groups, individuals	99	20.4%
	Victimised	Repeatedly targeted, a sense of powerlessness in relation to a more powerful other, individual, group, society	35	7.2%
Identity	Identity issues	Unstable sense of self, not knowing where one fits, what beliefs/preferences are	102	21%
	Seeks belonging	Active desire and attempts to fit into a group, even if this requires them to engage in activities against their beliefs/moral code	108	22.3%
	Seeks power	Seeks a position of power and influence over other people, seeks recognition/praise/admiration, seeks to avoid being the ‘one at the bottom’	138	28.5%
	Desire to intimidate others	Seeks to intimidate others in relationships, with violence, threats, or persona	152	31.3%
Mental health/complex needs	Personality disorder	Diagnosed personality disorder	18	3.7%
	Suicide/Deliberate self‐harm	History of deliberate self‐harm	94	19.4%
	ASD	Diagnosed autism spectrum disorder	48	9.9%
Personality traits	Vulnerable to exploitation	Evidence to indicate that they have been radicalised, evidence they have been persuaded to engage in harmful/criminal behaviours they wouldn't have initiated themselves	279	57.5%
	Low self‐esteem	Low view of self, lack of confidence	40	8.2%
	Impulsive/Thrill‐seeking	Risk taking & impulsivity	78	16.1%
Social network/Exposure	Violence exposure (historic)	Exposure to violence in the home or the community or possibly via extremist videos although we might need to keep this in mind as a separate code as and when it comes up	64	13.2%
	Family criminality	Family involved in crime	78	16.1%
	Associating with known others	Associating with individuals known to police/CVE	60	12.4%
	Extreme exposure	Exposure to extremism (online and/or offline), such as meeting known others, family members involved in extremism, exposure online to extreme narratives, extreme propaganda	95	19.6%
	Exploit peers	Exploited by current peer group	59	12.2%
	Gang association	Links to or involvements with gangs	86	17.7%
	Submissive/Bully/Leader	Bullying others	57	11.8%
	Lack of support	Lack of individuals to which that person can turn for support (services or family)	92	19%
Relationships	DV perpetrator	Perpetrator of physical, sexual, emotional or financial abuse	61	12.6%
	DV victim	Experiencing or witnessing physical, sexual, emotional abuse, between or from family members	39	8%
	Relationship instability	Lots of partners, an inability to keep a relationship going for any length of time, an inability to manage conflict, normal relationship difficulties	92	19%
	Relationship conflict	Lots of significant conflicts, fights, fighting in the streets, lots of calls by neighbours to the property where they live	115	23.7%
	Relationship breakdown	Important relationship (partner, family, friend) breakdown	82	16.9%
Trauma/Maladjustment	Bullying	Targeted by a group of individuals using direct or indirect behaviours resulting in actual or psychological harm	39	8%
	Poor engagement	Avoided/inconsistently engaged with mental health team, failed to engage in treatments, poor adherence to medication regime	21	4.3%
	Peer rejection	Didn't have many friends, experienced peer rejection (actively not included, pushed out) or neglect (not invited to anything, didn't fit in) spent most of childhood playing alone)	25	5.2%
	Peer delinquency	Hangs around with peers that are involved in antisocial or criminal behaviours/individuals with pro‐criminal or negative attitudes	32	6.6%
	Childhood abuse	Historic experiences of abuse, sexual, physical, emotional abuse or neglect as a child	69	14.2%
	Caregiver disruption	Disrupted care giver history, being in care, adopted, foster care, moved between family members, loss of primary caregiver e.g. mother died	104	21.4%
	Neglect	History of neglect, unloved, not enough food, uncared for	32	6.6%

### 3.4 Statistical Analysis

An overall network model was estimated to visualise the interactions between the included factors. The analysis proceeded in two phases. First, to answer ‘risk of what?’, we applied a community detection algorithm (Spinglass) to identify unknown communities (groups) of related risk factors. The results are groups of risk factors which researchers label based on interpreting the configurations of variables making up the discrete communities. Second, to answer ‘and why?’ we computed shortest paths to the extremist behaviour, extremist action. We focus only on action here as the purpose is to demonstrate the utility of a network approach for modelling the risk‐formulation process. It is possible to model pathways to all extremist behaviours, and indeed any risk factor in the network, however to do so would require far more space than the current manuscript allows.

#### 3.4.1 Network Estimation

We used the R package *bootnet* to estimate the network. Bootnet computes an undirected network graph and applies a Least Absolute Shrinkage and Selection Operator (LASSO) regularisation. The LASSO penalty results in a network which is both parsimonious and optimises goodness of fit. The regularisation employs a tuning parameter, where researchers can set values ranging from zero to one. A low value may suit more exploratory designs as it is more lenient, however may result in more spurious edges. A high value is more conservative, however may remove potentially important edges. We set the hyperparameter, gamma, to 0.15, which suits an exploratory research design. Further details can be found in Hevey (2018).

Lastly, we used the OR‐rule The OR‐rule is also more lenient, where only one non‐zero coefficient between two nodes is needed for a connecting edge to be drawn. The alternative, the AND‐rule, is more conservative, as it requires two non‐zero coefficients between nodes for an edge to be drawn. An edge connecting two nodes can be interpreted as a significant association, controlling for all other nodes in the network. Tutorial papers for implementing a psychometric network approach in R are widely available (Costantini et al. 2015; Epskamp, Borsboom, and Fried 2018).

The Spinglass algorithm (Pons and Latapy 2005) in the igraph package (Csardi and Nepusz 2006) was used to detect communities within the network. The algorithm is a hierarchical clustering algorithm which constructs communities based on random walks, where, on ‘short walks,’ the nodes you visit most often will be assigned to the same community. There are a range of community detection algorithms available. Some may be more suitable than others, depending on the research design. The Spinglass algorithm is largely effective at detecting communities among a small sample size, and so was implemented here. See Yang, Algesheimer, and Tessone (2016) for a full discussion of the different community detection algorithms.

#### 3.4.2 Network Stability

To compute the stability of the networks and all centrality indices, we bootstrapped 95% confidence intervals around the edge weights, and computed the edge‐weights difference test and the centrality difference test (Epskamp, Borsboom, and Fried 2018), via *bootnet*. The results are described in the Supporting Information S1.

## 4 Results

### 4.1 Overall Network Graph

**FIGURE 1:**
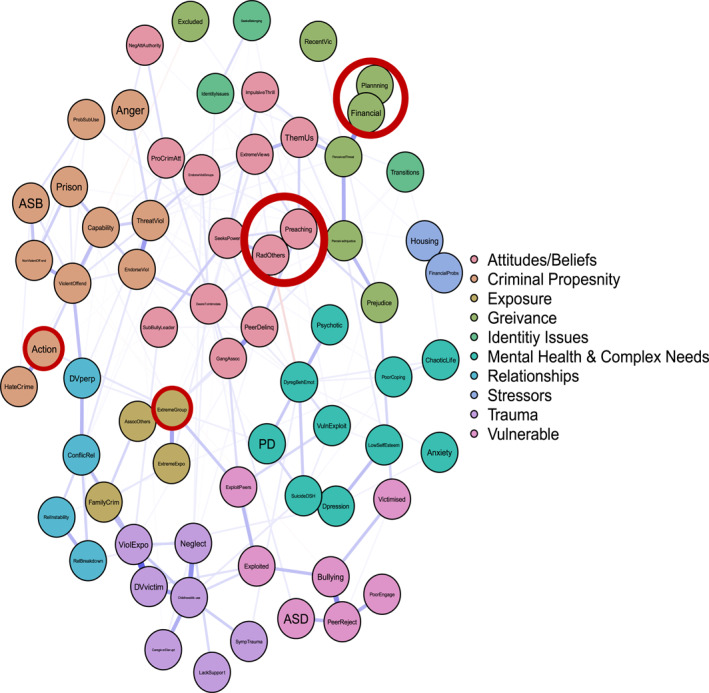
Overall network graph. Communities detected via the *Spinglass* algorithm and labelled by researchers. **Blue** edges between two nodes indicate a positive relationship. **Red** edges between two nodes indicate a negative relationship. The **thickness** of the edge relates to the strength of the relationship where thicker edges denote stronger relationships. The network is displayed so that nodes that are more related to one another appear closer together. Extremist behaviours highlighted in **red.**

Figure 1 presents the overall network graph and the results of the community detection algorithm. The network graph models the relationships between the 68 variables listed in Table 2. Mean edge weight was 0.03 and network density (the actual number of edges vs. the total possible number of edges) was low (7.77%), indicating a sparse network. Network accuracy and stability statistics were computed and are reported in full in the Supporting Information S1. Bootstrapped tests were performed with 2000 samples. Bootstrapped difference tests between non‐zero edges indicate the edge weights can be interpreted reasonably reliably (Supporting Information S1: Figure S2). Supporting Information S1: Figure S3 displays the bootstrapped confidence intervals of estimated edge weights. The results indicate some edge weights are more reliable than others, however, overall, the results suggest the edge weights are generally reliable.

Ten communities were identified via the Spinglass algorithm and were labelled by researchers interpreting the presenting pattern of variables. We describe each in turn below to answer ‘risk of what?’ Extremist behaviours are highlighted in Figure 1 in red.

#### 4.1.1 Attitudes & Beliefs

This community includes nodes predominantly related to attitudes, such as pro‐criminal attitudes, negative attitudes towards authority, us and them thinking, and beliefs such as extreme views, alongside personality traits such as impulsivity and thrill‐seeking, and a desire to intimidate others. This community also includes some exposure‐related factors, specifically related to exposure to delinquent peers and gang associations. In terms of extremist behaviours, this community includes radicalising others and preaching. This suggests that this constellation of factors is related to these types of extremist behaviours in our sample.

#### 4.1.2 Criminal/Violent Propensity

This community is characterised by a pattern of factors demonstrating a pre‐existing criminal/violent propensity. This includes active anger, problematic substance use, threatening violence, endorsing violence, anti‐social behaviour, non‐violent and violent offending, and committing hate crimes. Most notably, this configuration of risk factors includes the extremist behaviour action, suggesting that this pattern of factors is related to individuals in our sample engaging in extremist action.

#### 4.1.3 Exposure

The exposure community includes nodes related to exposure to crime and violence, including exposure to extremist groups. The configuration includes the extremist behaviour joining an extremist group.

#### 4.1.4 Grievance

This community is characterised by a pattern of factors related to grievance formation, including recent experiences of prejudice, perceived injustice, perceived threat, and recent victimisation. This cluster of factors includes the extremist behaviours financial support of terrorism and planning for an attack.

#### 4.1.5 Identity Issues

The identity issues community is a community of nodes defined by a period of transition, seeking belonging, and identity issues.

#### 4.1.6 Mental Health & Complex Needs

This community includes active symptoms of anxiety and depression, active psychotic symptoms, dysregulated behaviours and emotions, diagnosed or suspected personality disorder, low self‐esteem, poor coping skills, and a chaotic lifestyle.

#### 4.1.7 Relationships

The relationships community includes factors related to conflictual relationships, including relationship instability, relationship breakdown, and domestic violence perpetration.

#### 4.1.8 Stressors

This community includes nodes related to current stressors, including housing and financial issues.

#### 4.1.9 Trauma and Maladjustment

A pattern of childhood trauma and maladjustment characterises this community of nodes. These include childhood abuse, neglect, domestic violence victimisation, a lack of support, exposure to violence in the home, and active symptoms of trauma.

#### 4.1.10 Vulnerability

The vulnerability community includes nodes related to susceptibility to exploitation and victimisation. This includes diagnosed autism spectrum disorder (ASD), bullying victimisation, poor engagement with services, and being exploited by peers.

### 4.2 Pathways to Extremist Action

**FIGURE 2:**
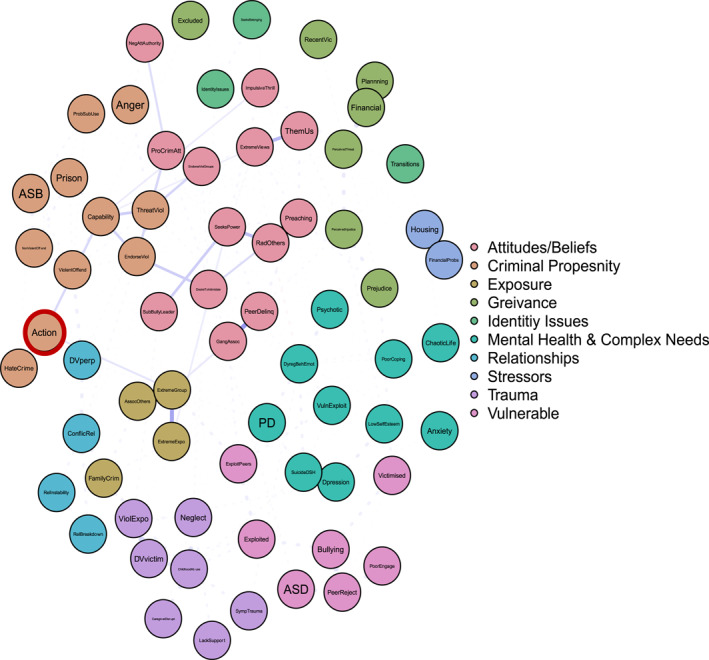
Shortest path from community ‘Attitudes/Beliefs’ to extremist behaviour ‘Action’. **Blue** edges between two nodes indicate a positive relationship. **Red** edges between two nodes indicate a negative relationship. The **thickness** of the edge relates to the strength of the relationship where thicker edges denote stronger relationships. The network is displayed so that nodes that are more related to one another appear closer together. Extremist behaviour **Action** highlighted in **red.**

**FIGURE 3:**
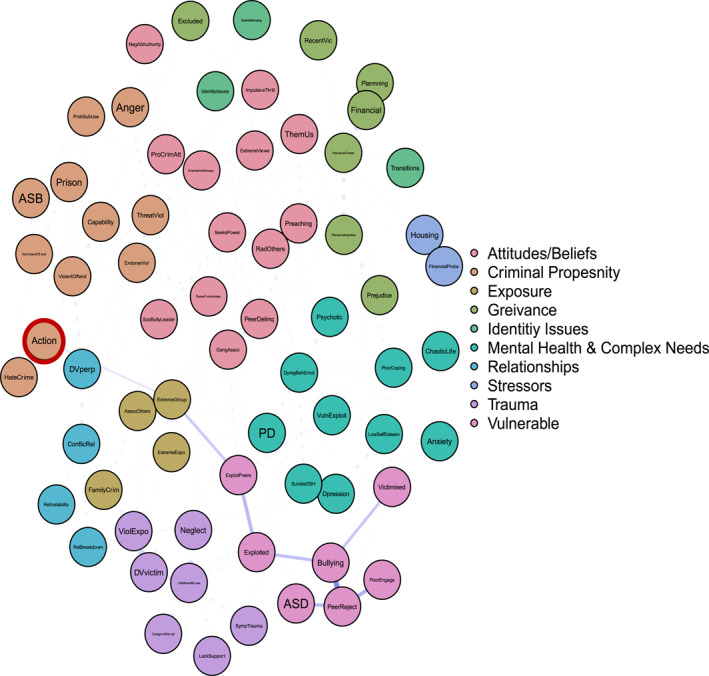
Shortest path from community ‘Vulnerable’ to extremist behaviour ‘Action’. **Blue** edges between two nodes indicate a positive relationship. **Red** edges between two nodes indicate a negative relationship. The **thickness** of the edge relates to the strength of the relationship where thicker edges denote stronger relationships. The network is displayed so that nodes that are more related to one another appear closer together. Extremist behaviour **Action** highlighted in **red.**

**FIGURE 4:**
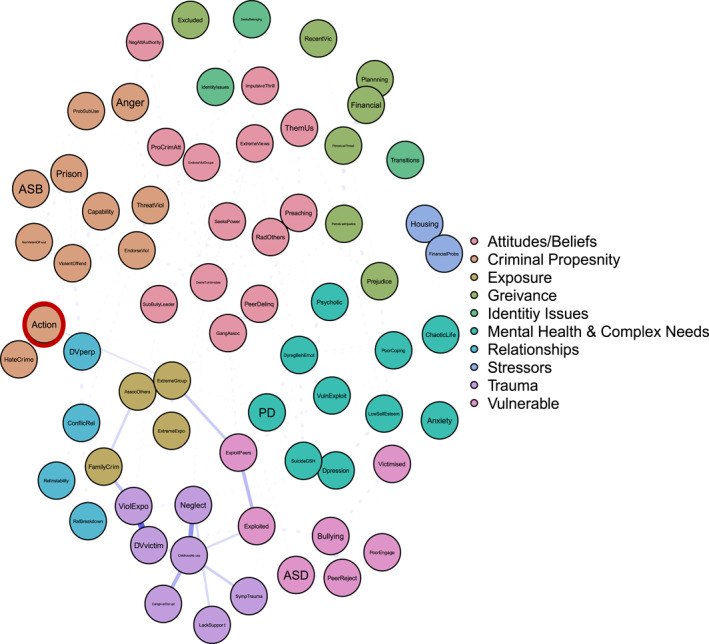
Shortest path from community ‘Trauma’ to extremist behaviour ‘Action’. **Blue** edges between two nodes indicate a positive relationship. **Red** edges between two nodes indicate a negative relationship. The **thickness** of the edge relates to the strength of the relationship where thicker edges denote stronger relationships. The network is displayed so that nodes that are more related to one another appear closer together. Extremist behaviour **Action** highlighted in **red.**

**FIGURE 5:**
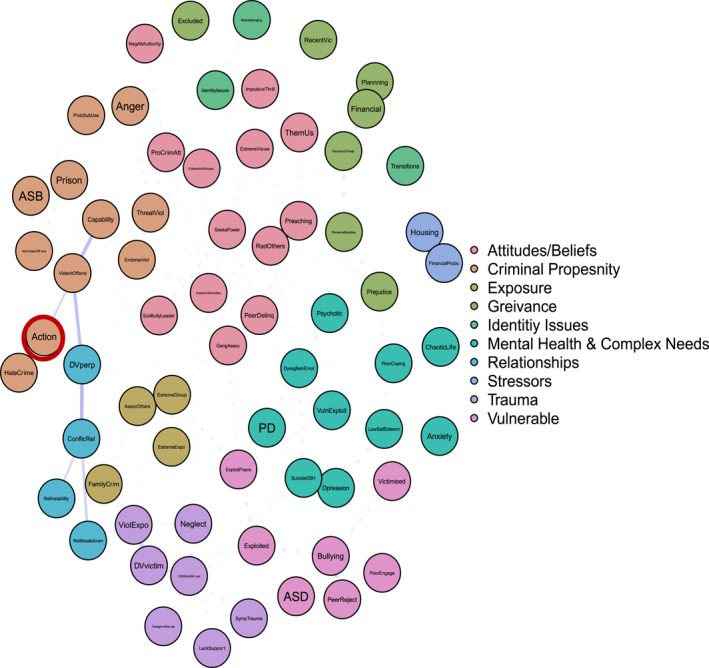
Shortest path from community ‘Relationships’ to extremist behaviour ‘Action’. **Blue** edges between two nodes indicate a positive relationship. **Red** edges between two nodes indicate a negative relationship. The **thickness** of the edge relates to the strength of the relationship where thicker edges denote stronger relationships. The network is displayed so that nodes that are more related to one another appear closer together. Extremist behaviour **Action** highlighted in **red.**

**FIGURE 6:**
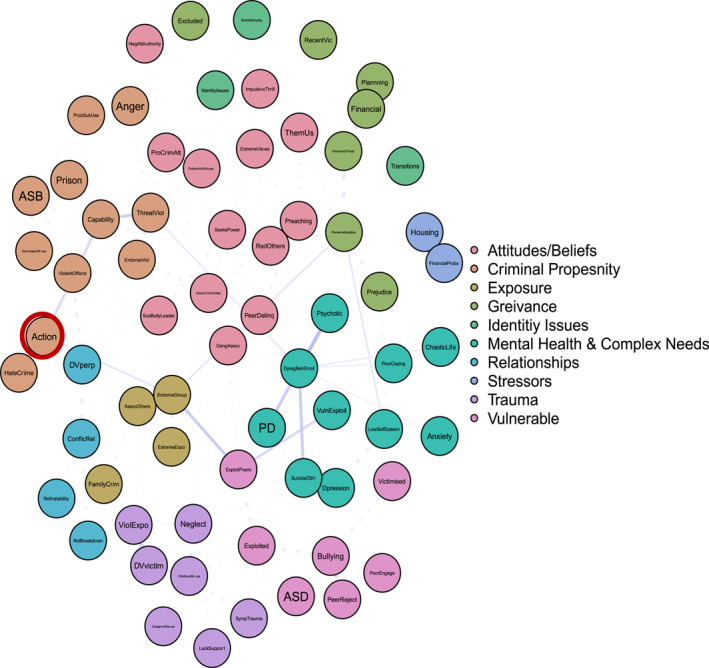
Shortest path from community ‘Mental Health & Complex Needs’ to extremist behaviour ‘Action’. **Blue** edges between two nodes indicate a positive relationship. **Red** edges between two nodes indicate a negative relationship. The **thickness** of the edge relates to the strength of the relationship where thicker edges denote stronger relationships. The network is displayed so that nodes that are more related to one another appear closer together. Extremist behaviour **Action** highlighted in **red.**

**FIGURE 7:**
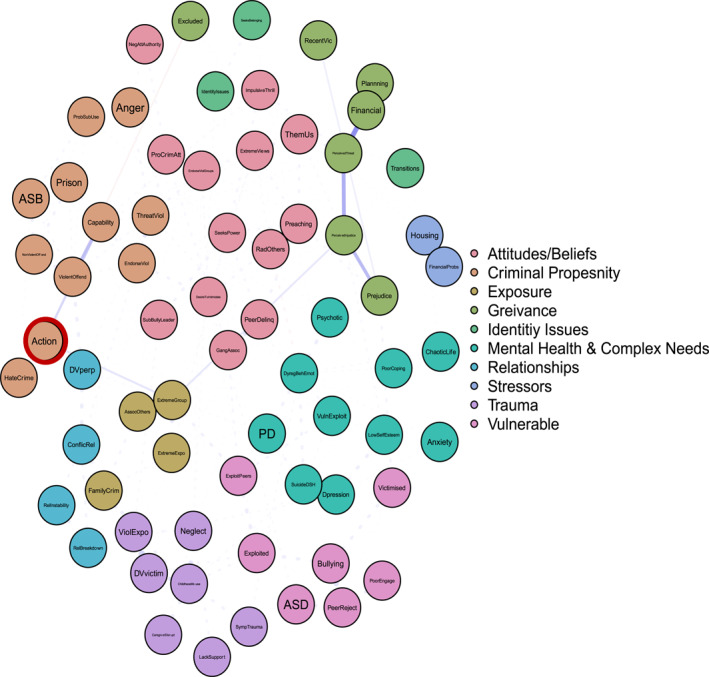
Shortest path from community ‘Grievance’ to extremist behaviour ‘Action’. **Blue** edges between two nodes indicate a positive relationship. **Red** edges between two nodes indicate a negative relationship. The **thickness** of the edge relates to the strength of the relationship where thicker edges denote stronger relationships. The network is displayed so that nodes that are more related to one another appear closer together. Extremist behaviour **Action** highlighted in **red.**

To answer ‘and why?’ Figures 2, 3, 4, 5, 6, 7 highlight pathways from the different communities of nodes to the extremist behaviour, action.

Figure 2 highlights the shortest paths from the ‘Attitudes/Beliefs’ community to action. It's important to note that these are the ‘shortest’ paths, in that they highlight the ‘quickest’ route from one set of nodes to another. This is only one possible route or pathway. Manually inspecting Figure 1 highlights the many multi‐final pathways to the various extremist behaviours, again highlighting the complexity of understanding why some come to engage in extremist behaviours.

First, Figure 2 demonstrates how exposure via criminal peers (peer delinquency and gang association) relates to exposure to extremism and extremist group membership. Extremist group membership in turn is related to extremist action. This demonstrates a ‘route’ to extremist action driven in part by exposure, both to criminality and extremism.

Second, Figure 2 highlights how the extremist behaviours preaching and radicalising others relate to extremist action via a desire to intimidate, seeking power, and being a bully/leader. This cluster of risk factors in the presence of threatening and endorsing violence, and capability (including violent offending) relates to extremist action. This pathway highlights a pattern of risk characterised by both seeking power and a desire to intimidate, coupled with concerning extremist behaviours (preaching and radicalising others) which demonstrates an extremist ideology, and manifests in violence and extremist action.

Finally, Figure 2 highlights how impulsivity and pro‐criminal attitudes, including endorsing violent groups, alongside extremist views and them and us thinking, leads to threatening and endorsing violence, capability (including violent offending), and in turn relates to extremist action. This pathway is more characteristic of a general pattern of offending interacting with extreme views to result in extremist action.

Figure 3 highlights the shortest path from the vulnerability community to the action. The pathway highlights how a pattern of bullying, peer rejection, and victimisation relates to being exploited by peers. Via exploitation this pattern of risk factors is related to joining an extremist group, which in turn relates to extremist action. This pattern also includes diagnosed ASD, highlighting one way in which ASD could related to extremist action, for some.

Figure 4 shows how trauma and maladjustment can relate to extremist action. First, Figure 4 highlights a pathway characterised by domestic abuse victimisation, exposure to violence in the home, and family criminality which leads to associating with known (extremist) others, extremist group membership, and extremist action. This pathway demonstrates a route to action underpinned by a violent home and familial criminality, leading to exposure to extremists and engaging in extremist action. Second, Figure 4 demonstrates a route to action via trauma and maladjustment—child abuse, neglect, and lack of support—and exploitation, again leading to extremist group membership and extremist action. This pathway is characterised by a susceptibility driven by childhood trauma and a vulnerability to exploitation.

Figure 5 demonstrates how conflictual relationships and domestic violence perpetration interacts with violent offending and capability, leading to extremist action. This pathway is characterised by a propensity for both ‘public’ and ‘private’ violence where domestic abuse perpetration may be an indicator of risk and capability.

Figure 6 highlights pathways from mental health and complex needs to action. Several routes are evident. First, low self‐esteem and perceived injustice interact with delinquent peers and extremist group membership, leading to extremist action. Here, group membership may act as resolution for low self‐esteem and perceived injustice, leading to extremist action. Second, mental health, complex needs, and poor coping styles lead to dysregulated behaviours and emotions, resulting in threatening violence. When coupled with capability and violent offending, they lead to extremist action. This pathway demonstrates one way poor mental health can drive engagement in violent extremism.

Finally, as previously described, there is a pathway characterised by vulnerability to exploitation, peer exploitation, and extremist group membership, leading to extremist action. Here, again, it is evident that the underlying driver of action in these instances is a vulnerability to exploitation.

Lastly, Figure 7 describes pathways between the Grievance community and action. Here, grievance appears to be a motivation to action where prejudice, perceived injustice, perceived threat, and recent victimisation relate to mobilisation indicators including attack planning and financial support of terrorism, interacting with extremist group membership and leading to extremist action.

## 5 Discussion

The present study aimed to introduce a methodology capable of modelling the complexity that practitioners deal with day‐to‐day when assessing and managing susceptibility to violent extremism. We applied psychometric network modelling to the data generated from the case files of approximately 500 individuals who were assessed as presenting some sort of CT concern. To answer ‘risk of what?’, we disaggregated violent extremist outcomes and detected 10 communities that each involved a cluster of risk factors and for some, particular outcome behaviours. To answer ‘and why?,’ we examined different routes to concerning behaviour, using extremist action as an example. Broadly speaking the results provide a ‘proof of concept’—that it is possible to generate empirical evidence to support an SPJ approach to violent extremist risk assessment and management. More specifically, our results highlight that there is value in disaggregating outcomes to better understand presenting risk. We highlight different risk profiles associated with each of the different extremist behaviours in an upstream P/CVE cohort, reiterating the need to be explicit in understanding risk of what (Borum 2015b; Roberts and Horgan 2008; Logan 2021).

In terms of the criminal/violent propensity profile, the identified pattern of indicators present as typical of more general violent offenders. These include anger, violent threats and attitudes, various violent and non‐violent offending types, and problematic substance use. Anger has long‐established associations with violence and offending of many forms, summarised by Loza and Loza‐Fanous (1999). The same is true of substance misuse (Boles and Miotto 2003; Friedman 1998). Previous offending is also linked to later violent, and more severe violent offending (Suonpää, Kivivuori, and Aaltonen 2018; Tärnhäll et al. 2023). More generally, this relates to the common criminological finding that a very small proportion of the population have disproportionate and lifelong violent, antisocial, varied, and severe criminal careers, and are often characterised by substance abuse and poor emotional regulation among other psychological and personality factors (Moffitt 1993; Vaughn et al. 2011, 2014).

Overall, the criminal/violent propensity community similarly reflects a risky pattern of factors for many forms of violence and offending, including extremist action. This is perhaps unsurprising given that previous criminality and violence is consistently identified as a predictor of extremist action (Wolfowicz et al. 2021). The violent extremism research and practice fields often assume violent extremism is a fundamentally different behaviour to general violence due to the ideological component, meaning theoretical frameworks, empirically established risk factors, and successful interventions cannot be transferred across, and tailored approaches are required. Our findings here, however, suggest that for some, risk of violent extremism is better understood within the context of violence and severe offending, than overemphasising an ideological component. Perhaps, therefore, these individuals would benefit from what we know works in general violence risk management, and less from ideologically‐based interventions.

Mobilisation indicators, financial support of extremism, and attack planning were associated with a profile suggesting these behaviours can be grievance‐driven. Grievance is often conceptualised as a motivation to action (Borum 2014; Brooks and Barry‐Walsh 2022; McCauley and Moskalenko 2014; Pathé et al. 2018). For instance, Horgan (2008) suggests grievance can be a ‘push’ factor motivating individuals to engagement in violent extremism. Preaching and radicalising others demonstrated strong associations with extremist and pro‐criminal attitudinal risk factors, alongside personality factors such as seeking power and a desire to intimidate. This suggests the behaviours are driven in part by attitudes and beliefs alongside a desire to control and/or influence others. Finally, extremist group membership is associated with a risk profile characterised by exposure to violent extremism. Exposure is consistently highlighted as an important and influential mechanism for understanding susceptibility to violent extremism (Clemmow, Bouhana, and Gill 2020; Clemmow et al. 2023; Hassan et al. 2018; Pauwels, Brion, and De Ruyver 2014; Pauwels and Schils 2016; Schumann et al. 2023).

Shortest path analysis further identified different pathways from the risk profiles to the extremist behaviour, action. In general, the results reiterate the multi‐finality of pathways to engagement in violent extremism (Corner, Bouhana, and Gill 2019; Gill, Farnham, et al. 2021). Our findings reiterate the need to build empirical evidence which speaks to understanding the what, why, who, and how of engagement in violent extremism (Bouhana and Schumann 2022) to better inform risk assessment and management practice.

For instance, the role of mental health in pathways to violent extremism is not well understood (Corner and Gill 2015). Research has moved on from describing violent extremists as either mentally disordered or not, but we continue to lack the understanding to articulate when and for whom poor mental health contributes towards susceptibility to violent extremism. Our results identify how poor mental health and complex needs **can** be relevant to susceptibility to violent extremism, **for some**. For instance, Figure 1 articulates pathways which demonstrate how dysregulated behaviours and emotions relate to threats of violence and can lead to extremist action. However, equally, the same risk factor demonstrates a protective effect against radicalising others, suggesting that those presenting as dysregulated are less likely to radicalise others. In another instance, depression, suicide attempts, and deliberate self‐harm relate to childhood trauma, maladjustment, and a susceptibility to exploitation, which relates to extreme group membership and extremist action. However, there is no direct route from poor mental health and complex needs to extremist action. In other words, it is the interaction of poor mental health and complex needs with a range of other factors including attitudes, behaviours, situational influences, and exposure, which culminate in susceptibility to violent extremism, not simply the presence of poor mental health or diagnosed mental illness.

This is true too for the plethora of risk factors which correlate with violent extremism. These direct relationships do little to explain risk of what and why and therefore do not provide the science that practice needs to evidence an SPJ approach to violent extremist risk assessment and management. This is especially true considering that the cumulative and interactive effects of different combinations of risk factors often change the strength or nature of the observed direct effects (Clemmow et al. 2023). Hence a key message of the present study is to add to the call for research to continue to advance towards understanding the multifactorial nature of risk and protective factor interactions, to better understand how violent extremist risk emerges. As previously stated, the SPJ approach is generally regarded as best practice in this space, as a formulation‐based approach to risk assessment and management provides a framework for organising and understanding the complexity practitioners are confronted with, over and above the capabilities of an actuarial tool, for instance. In the following section we discuss the practical implications of our findings.

### 5.1 An SPJ Approach

An SPJ approach to risk assessment is seen as a healthy compromise between overly structured and prediction‐focused actuarial instruments, and unstructured clinical judgments. It involves using evidence‐based risk factors to structure the information gathering and assessment process, but crucially relies on clinical judgement to understand the relevance of risk factors to a particular case, and form an overall formulation of behaviour and risk (Borum 2015a; Dean and Pettet 2017; Hart and Vargen 2023; Sarma 2017). While the SPJ approach is regarded as best practice in violent extremist risk assessment, there are currently several obstacles to the effective use of SPJ in this field. These can be largely summarised as a need for (1) case formulation, (2) identification of evidence‐based risk factors and a framework to understand them, for different subgroups, and (3) specification of the outcome of concern. Our findings correspond to each of these obstacles, providing further evidence that adopting an SPJ approach for risk assessment and management susceptibility to violent extremism is both possible and preferable.

#### 5.1.1 Clinical Judgement and Case Formulation

Firstly, to harness its full strengths, SPJ approaches require using clinical judgement to develop an overall case formulation: an explanatory framework of an individual's motivations, justifications, objectives, precipitators, and overall decision‐making, complemented with respective risk management directives and scenario planning (Gill et al. 2023; Hart and Vargen 2023; Sarma 2017). This formulation approach is currently lacking in existing commercially‐oriented SPJ instruments for violent extremism (Sarma 2017). The findings in this paper, however, highlight its potential and significance. Historically, (actuarial) risk assessment instruments have involved aggregating the presence of individual risk factors to reach a cumulative score. This assumes a linear relationship with risk, which may be true of other violence forms but not of violent extremism (Borum 2015a; Sarma 2017). Our findings instead show that risk factors are interdependent, not all equally relevant to a case, and organised in clusters/communities that are related to different outcomes. This reflects the necessity of an SPJ approach that appreciates the complexity and multivariate nature of risk, and where it is not aggregated risk factors that determine an assessment, but their relevance and interdependency (Borum 2015a; Gill et al. 2023; Sarma 2017). Clinical intuition, experience, and expertise is required to understand this complex interdependency for an individual case and build a formulation around the relevant risk factors, to understand the ‘why’. Altogether, this gives room for evaluators developing individual case formulations to understand the nature of their risk for a certain behaviour at a certain time, which can then guide assessment and management (Borum 2015a, 2015b; Gill et al. 2023).

#### 5.1.2 Identifying Evidence‐Based Risk Factors

Alongside clinical judgement, SPJ instruments also require evidence‐based risk factors to structure the information‐gathering and assessment process (Hart and Vargen 2023). The use of SPJ in P/CVE has therefore been criticised due to the insufficient empirical evidence base for individual risk factors for terrorism involvement (Borum 2015a; Sarma 2017). Indeed, the general justification for using completely unstructured clinical judgment is that the complexity of risk is such that we are forced to rely on clinician judgement alone (Hart and Vargen 2023). For violent extremism, research and understanding of the complexity of risk and the heterogeneity of cases and radicalisation pathways is in its infancy, breeding concerns that it is not possible to base a risk assessment in any way on empirically derived factors (Borum 2015a). However, our findings show that by using a network approach, and by considering risk factors as interdependent rather than standalone, it is possible to empirically analyse and visualise the way certain risk factors are related to susceptibility to violent extremism. While complex, there are still patterns and dynamics that can be empirically identified and analysed to provide a structure for gathering information and assessing risk in this space.

#### 5.1.3 Outcome Specification

Finally, many argue that a current failure of SPJ approaches to violent extremist risk assessment is an inability to specify the outcome that is being assessed (Borum 2015a, 2015b; Gill et al. 2023; Roberts and Horgan 2008; Sarma 2017). Here we have shown that it is possible, and useful, to specify the outcome. Our analysis reveals that communities of interdependent clusters of risk factors are relevant not to violent extremist risk overall, but to individual outcome behaviours (e.g., ‘attitudes/beliefs’ housing radicalising others and preaching, vs. ‘exposure’ housing joining an extremist group). Disaggregating by behaviour therefore provides a way to structure and visualise risk. The resulting clusters of risk factors for different behaviours are distinct, but use of path analysis also conceptualises the feasible routes and pathways between them. This provides scope for scenario planning, where the overall case formulation can be linked to different outcomes, and their relative likelihoods and precipitating factors or situations (Hart and Logan 2011; Sarma 2017). For example, Figure 2 provides an indication of what might need to happen, or what risk factors would have to be accumulated, for an individual demonstrating the ‘attitudes/beliefs’ risk profile and at risk of radicalising or preaching, to move towards extremist action.

#### 5.1.4 Limitations

It's important to acknowledge the limitations of the present study, particularly with respect to any practical implications. First, the Channel program is voluntary and so the data may be subject to selection effects, where people who hold more pro‐social views may be more likely to consent to the process, and vice versa. Hence the data may not be a true reflection of the population we intend our findings to generalise to—individuals susceptible to violent extremism.

Second, the data are from 2012 to 2014, and the threat landscape has undoubtedly changed over the last 10 years. Whilst we believe the underlying mechanisms driving susceptibility to violent extremism largely remain constant, the indicators of those mechanisms might look different. For instance, exposure to extremism is still key to understanding susceptibility to violent extremism, however the role of the internet, AI, gaming platforms, and so on, are potential new indicators of exposure to extremism, which would not have existed in 2012. However, the broader aim of the paper was to demonstrate an approach to take forward to begin to make sense of the complexity practice routinely deals with, and build data‐driven evidence for an SPJ approach to assessing and managing violent extremist risk. Despite the limitations of the data, there is still value in this initial proof of concept.

Third, this a secondary analysis of data previously collected by a different team. The research team did not have access to original source material, however one of the authors did and so was able to clarify concerns and/or consult source material where needed. Finally, our analytical approach is explicitly exploratory, and any findings should feed into theory development and/or hypothesis generation for testing with confirmatory research designs.

## 6 Conclusion

Our findings provide a way of structuring assessments: clusters of risk factors are related to different outcome behaviours (risk of what), and there are potentially predictable routes through these between behaviours (and why). This reveals that within the complexity of violent extremist risk, there is both the potential for and value in: clinical judgement through a formulation approach to understand this complexity in the individual case; uncovering empirically valid clusters of risk factors; and disaggregating outcome behaviours within susceptibility to violent extremism. We recommend that SPJ instruments and procedures should continue to be developed in this space, with a focus on accommodating this interdependency of risk factors for different outcome behaviours.

The focus on individual risk factors, rather than their summative or interactive effects, is insufficient for practice (see Clemmow et al. 2023). The increased publicity around a single risk factor (e.g., mental health disorders, or domestic abuse, etc), has a knock‐on effect up‐stream where referrals disproportionately reflect the in‐vogue risk factor of the time, creating a referral bias, potentially a later confirmatory bias, and then an almost guaranteed stigma amongst groups of people nowhere close to posing an extremist risk.

Although the approach taken here is purely inductive, the resulting clustering of factors intuitively lines up with several well‐established psychological and criminological theories about the developmental pathways of (extremist) violence. This also matters for SPJ guidance going forward because theories help organise assumptions, make sense of difficult phenomena, and—in combination with empiricism—anchor formulations (Gill and Rottweiler 2023). Most importantly, our clustering of factors shows that no one theory will have explanatory power for all cases. Rather, it requires a consideration of what theory is hypothesised as the best fit for the individual case being assessed. This comes down ultimately to the training and skills development of risk assessors to have a broad knowledge of both the ever‐developing field of theory and empiricism. Our results provide a blueprint to help assessors define the theory or theories that best suits their individual case or cohort of cases. Much more work is needed to understand whether certain segments of our population (e.g., males vs. females, youth vs. adults) are more susceptible to certain concatenations of factors.

Of course, the majority of this paper has been focused on the risk assessment component of the puzzle. Risk management is the other side of the coin, about which there is much less written or tested. The subgroups identified in this paper suggest strongly that narrow interventions which assume the same intervention strategy will suit all onboarded cases once they meet some sort of threshold of risk are likely to be less effective than bespoke interventions developed for the individual's constellations of factors and drivers. Our results should inform which types of interventions will suit which types of cohorts. This may require a concerted effort to think about who the deliverer of such interventions should ultimately be, and identify gaps in current service delivery across different national contexts.

Notwithstanding issues related to accessibility (e.g. waiting lists) and individual responsivity to intervention, this might require leaning on existing services ostensibly designed to manage other forms of harmful behaviour who are not used to managing this specific form of risk. However, the interventions they use for the cohorts they deal with already might be much more suitable than others designed specifically as P/CVE interventions. Emphasis should be placed on *might*. In a field where evaluations are few and far between, especially in Western contexts, we really do not know what works. We certainly do not have the evidence base to know what works for specific clusters of risk factors. But the empirics are clear; one‐size fits all approaches are likely not the best route as the determining factors are too diffuse, and the extremist outcomes too varied.

## Author Contributions

**Caitlin Clemmow:** conceptualisation, data analysis, write up. **Nicola Fowler:** data generation, data analysis, write up. **Amber Seaward:** data analysis, write up. **Paul Gill:** funding, write up.

## Ethics Statement

The study was a secondary analysis of data collected by a team of practitioners and so no human subjects were involved. The practitioner team originally collated the data from case files and so no human subjects were involved at the primary data generation stage, either. The study was approved by UCL Research Ethics team on 24th August 2017, under the project title ‘Gauging the Risk of Incidents of Extremist Violence Against Non‐Combatant Entities (GRIEVANCE).’

## Conflicts of Interest

The authors declare no conflicts of interest.

## Supporting information

Supporting Information S1
