# Supplementary material for: Risk of What and Why? Disaggregating Pathways to Extremist Behaviours in Individuals Susceptible to Violent Extremism
Source: Behav Sci Law. 2024 Dec 8;43(2):228–47. doi: 10.1002/bsl.2710 (PMC11961345; doi:10.1002/bsl.2710)
Supplement: Supplementary file 1 — Supporting Information S1 [file BSL-43-228-s001.docx]

Figure S1. Age distribution

Figure S2. Bootstrapped difference test between non-zero edges. Coloured squares represent edges, ordered from highest (darkest, see top right corner) to lowest (lightest, see bottom left corner) edge weight. Black squares indicate significant difference between edges (p < .05). Grey squares indicate no significant difference. Axis have been removed to avoid cluttering.

Figure S3. Bootstrapped confidence intervals of estimated edge-weights for the Overall network (Figure 1). *Red line* indicates sample values. *Grey area* indicates bootstrapped 95% CI. Edges are ordered from highest (top) to lowest (bottom) edge-weight. Node labels are removed to avoid cluttering.

Figure S4. Average correlations between centrality indices of networks sampled with persons dropped and the original sample. Stability of centrality indices can be quantified by the CS-coefficient. Strength CS(cor = 0.7) = 0.284. Acceptable values should not be below 0.25.
